# Effect of Styrene-Diene Block Copolymers and Glass Bubbles on the Post-Consumer Recycled Polypropylene Properties †

**DOI:** 10.3390/ma13030543

**Published:** 2020-01-23

**Authors:** Maria Râpă, Bogdan Norocel Spurcaciu, George Coman, Cristian Andi Nicolae, Raluca Augusta Gabor, Paul Niculae Ghioca, Andrei Constantin Berbecaru, Ecaterina Matei, Cristian Predescu

**Affiliations:** 1 Center for Research and Eco-Metallurgical Expertise (ECOMET UPB), University Politehnica from Bucharest, 313 Spl. Independentei, 060042 Bucharest, Romania; rapa_m2002@yahoo.com (M.R.); g.coman@hotmail.com (G.C.); andrei_berbecaru@yahoo.com (A.C.B.); cpredescu56@yahoo.com (C.P.); 2 National Institute for Research & Development in Chemistry and Petrochemistry (ICECHIM), 202 Spl. Independentei, 060021 Bucharest, Romania; ca_nicolae@yahoo.com (C.A.N.); ralucagabor@yahoo.com (R.A.G.); pghioca@yahoo.com (P.N.G.)

**Keywords:** recycled polypropylene, block copolymers, glass bubbles, processability, mechanical tests

## Abstract

The recycled polypropylene (rPP) materials that meet technical requirements such as reducing the dimensions and improving the tensile, elongation, impact strength, thermal stability, as well as melt processing, are required for the manufacturing industry. In this paper, we studied the mechanical and thermal properties of post-consumer rPP by adding both synthesized thermoplastic elastomers, and glass bubbles (GB) by a melt allowing process. Styrene-butadiene (SBS) and styrene-isoprene (SIS) block-copolymers that had a styrene content of 30 wt% were synthesized by anionic sequential polymerization. The obtained post-consumer rPP composites were characterized by optical microscopy, scanning electron microscopy (SEM), mechanical analyses (tensile, density, hardness, VICAT softening temperature (VST), heat deflection temperature (HDT), dynamic mechanical analysis (DMA), IZOD strength) and thermal analyses (differential scanning calorimetry (DSC) and thermogravimetric analysis (TGA)). Weight reduction and improvement of the tensile, elongation, impact strength, thermal stability, as well as melt processing of post-consumer recycled polypropylene (rPP) properties compounded with thermoplastic elastomers and glass bubbles, sustain the use of these formulations for engineering applications.

## 1. Introduction

Polypropylene (PP) ranks third in the plastic manufacturing field [1], being intensely processed for auto components, fittings, pipes, cables, packaging, electronic items and fibers for concrete reinforcing. Generally, PP shows a low impact, especially at low temperatures, and low strength. In order to overcome these deficiencies, as well as to reduce the cost of the final material, various additives and fillers are added during PP processing, such as talc, CaCO_3_, glass beads [2] glass fibers [3,4], clay [5,6], carbon particles, coupling agent (maleic anhydride), wood flour [7], antistatic agents [8], stabilizers [9], having the main role of increasing the stiffness and impact resistance. For example, by adding titanate coupling agent and talc (30%–40%), the melt flow index and toughness of PP increased [10]. Thermoplastic elastomers, such as ethylene propylene-rubber (EPR) [11,12], ethylene–propylene–diene (EPDM) [13], poly(styrene-b-ethylene/butylene-b-styrene) (SEBS), poly(styrene-b-butadiene-b-styrene) (SBS) [14] are also used for the melt processing of PP due to their compatibilizers and thermoplastic properties [15]. It has been documented that the blends of PP with thermoplastic elastomers are immiscible in melt processing and led to a lower interfacial adhesion [12].

The intensive use of PP goods led to a huge amount of waste, both from the technological process and the post-user consumer, reaching 25.8 million tons in 2014 [16]. A smart approach to the recycling of post-consumer PP items envisages both to transform them into new consumer items and to protect the environment. As recycling methods for PP, the following are known—primary recycling, mechanical (secondary recycling), chemical and pyrolysis methods [17,18,19]. Among these methods, the secondary recycling is preferable for reducing the amount of plastic waste, both from economic and ecological perspectives.

The recycled neat post-consumer polypropylene showed a noticeable decrease in the mechanical and impact properties with a number of processing cycles in the melting state, which occurs as the thermo-mechanical degradation induced by the chain scission mechanism of PP [20,21]. For example, compared to the virgin material, the addition of recycled PP causes a decline in the crystallinity degree, expressed in low viscosity [22]. Usually, up to 50 wt% post-consumer recycled polypropylene is added to virgin polymeric matrix processed by extrusion and injection technologies [23]. However, due to the presence of polyethylene as discontinuous phases in a polypropylene continuous phase [24], the thermal and mechanical properties of post-consumer recovered items are weaker compared to those of unmodified polypropylene [1]. To overcome these limitations, reinforcing agents and compatibilizers have been introduced into the recycled PP matrix during the melt mixing process to manufacture refrigerator plastics [25], 3D printing materials [26], auto components [27], and so forth.

The literature has noted the use of low cost fillers, such as natural fibers [28], glass fiber [29], wood flour [30], talc, montmorillonite [21,31] for improving the mechanical properties of post-consumer recycled PP, in the context of sustainable development. It is well known that the mechanical tests of post-consumer recycled PP are dependent on the nature of additive and filers, their size (preferably less than 5 μm), the ratio aspect close to unity, the homogeneous dispersion in polymer matrix, and the possible interactions between additives [32]. The most used fillers for rPP processing increased the density, tensile and flexural strength properties. Therefore, the introduction of 30 wt%–35 wt% glass fiber into the PP compound led to an increased density (1.17 g/cm^3^) [29] compared with PP, while the addition of 15 wt% glass fibers to the rPP composites led to increased tensile strength by 70% [4]. Wang [21] reported the increase in viscosity of PP composites containing talc. Inácio [33] studied the properties of a post-consumer recycled PP/EPDM/talc compounded with bamboo fiber. In the presence of compatibilizer and bamboo fiber, only the tensile, flexural strength and fatigue life increased, while the impact strength and elongation at break dramatically decreased. The recycled polypropylene materials that meet the technical requirements, such as reducing the dimensions, improving the tensile, elongation, impact strength and thermal stability, as well as melt processing, are required for the manufacturing industry. The improved properties of recycled PP (tensile strength, impact strength) could be obtained by adding the long chain branched structure such as glycidyl methacrylate (GMA) through reactive extrusion [34], or chemical initiators such as peroxide and styrene and peroxydicarbonate (PODIC) [35].

Our team has investigated the use of synthesized and commercial thermoplastic elastomers (TPEs) to obtain post-consumer recycled polypropylene composites with improved properties [36,37,38,39]. TPEs consist of elastomeric (butadiene/isoprene) blocks giving the cross-linked rubbers behavior and styrene blocks that confer thermoplastic properties at processing temperatures. In the case of melt processing of incompatible polymers or with limited compatibility, as in the case of TPEs and polyolefins, it has been shown that the uniform dispersion and the optimum dimensional of elastomeric phase is most likely obtained when the melt viscosity at the modification temperature of the components is as appropriate as possible [37]. This optimal dispersion of the elastomeric phase in the amorphous polypropylene matrix leads to better energy absorption, a more uniform dispersion of internal stresses and, thus, more efficient prevention of the propagation of the micro-cracks when the material is subjected to mechanical shocks. Based on our previous results, the aim of this study was to obtain block-copolymers with a content of 30 wt% styrene and molecular masses around 100,000 g/mol, which show a melt viscosity closest to that of recovered polypropylene. Despite the fact that TPEs are expensive, they are commercially used to improve the impact properties of rPP composites due to the significant increase of elongation and impact. Taking into account that the recycled polypropylene price represents ~40% from that of virgin PP, and the block copolymers have been introduced in the amount of 10%–20%, the final price with raw materials will be ~60% of that of virgin PP. This estimation, together with the superior impact and endurance properties of rPP composites demonstrate the feasibility of including block copolymers in rPP for large scale operations. At the same time, it must be emphasized that by this method, the polypropylene waste is recovered, thus contributing to ecological and environmental depollution.

Among styrene block-copolymers used to impact the properties of rPP, it was reported that the introduction of SEBS led to a significant rise in the elongation (19 times higher when compared with unmodified post-consumer recycled PP) and IZOD impact (10 times higher) than SBS (elongation at break increased 4 times higher and notched Izod increased 2 times than recycled PP) [40]. By increasing the elastomer content, the elongation and impact strength increased, while the tensile strength and hardness reduced [14,39,40]. However, an optimal content of elastomer in recycled polyolefin should be selected, taking into account the balance between toughness and stiffness. In order to extend the effect of elastomer on the rPP composites, our team also added an inorganic filler and glass bubbles.

In the present paper, the design of composites based on post-consumer recycled polypropylene (rPP), synthesized styrene-butadiene/styrene-isoprene block-copolymers with 30 wt% styrene content (SBS and SIS, respectively) using the anionic sequential polymerization and glass bubbles (GB) by melt allowing, and their processability, mechanical and thermal properties, are analyzed. These post-consumer recycled PP/elastomer/GB composites were characterized by optical microscopy, processability on a Brabender Plastograph, mechanical measurements (density, tensile properties, dynamic mechanical analysis (DMA), hardness, impact), and thermal analyses (differential scanning calorimetry (DSC), thermogravimetric (TG), heat deflection temperature (HDT), and VICAT softening temperature). These analyses revealed that by introduction of SBS or SIS block copolymers and glass bubbles into rPP, the additional properties to impact strength and elongation were obtained in comparison with previous studies, in terms of improving density and melt processability.

## 2. Materials and Methods

### 2.1. Synthesis of SBS/SIS Block-Copolymers

The styrene-butadiene and styrene-isoprene block-copolymers were obtained using one of the most modern techniques applied in polymer synthesis, namely sequential anionic polymerisation of monomers using cyclohexane solution (1.5 mol), in the presence of n-butyl lithium as initiator [41]. The anionic polymerisation permits the precise control of the molecular weight and the formation of styrene-diene block-copolymers with well-defined linear and branched structures. The above mentioned facilities require keeping the anionic polymerisation active centers’ concentration constant during the synthesis through a three-stage process. This procedure is named “living” polymerisation.

In the first stage, the synthesis of the polystyrene block is performed by the styrene polymerization, initiated by n-butyl lithium. The molecular weight is adjusted by the initiator dosage. As a result of styrene polymerisation, the active (“living”) polystyrene chains will be formed as polystyryl-lithium.

In the second stage, the synthesis of the polybutadiene (or polyisoprene) block is carried out by adding a calculated amount of butadiene (or isoprene) to polystyryl-lithium. In this case, polystyryl-lithium plays the role of polymerisation initiator of butadiene (or isoprene) and the latter will polymerise to polystyrene chains, resulting in the active diblock-copolymer of polystyrene-polybutadienyl-lithium (or polystyrene-polyisoprenyl-lithium).

**Figure 1:**
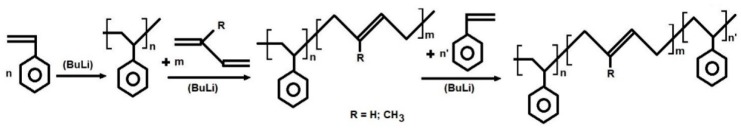
Synthesis of Styrene-butadiene (SBS) (R = H) and styrene-isoprene (SIS) (R = CH_3_) block copolymers by living polymerization.

In the third stage, in order to obtain linear block-copolymers, a new calculated amount of styrene, equal to that used in the synthesis of the first polystyrene block, is introduced to react with the active diblock-copolymer of polystyrene-polybutadienyl-lithium (or polystyrene-polyisoprenyl-lithium), resulting in styrene-butadiene (or isoprene) triblock-copolymer (Figure 1).

For this study, a starred SBS block copolymer and linear SIS block copolymers were synthesized. In the case of the synthesis of starred block-copolymers that had four arms, a coupling agent was introduced into the system (usually silicon tetrachloride) which reacts with the active diblock copolymer of polystyrene-polydienyl-lithium. To stabilize the synthesized block copolymers, 1% 2, 6-di-tert-butyl-4-methylphenol (Topanol OC) was used. The obtained block copolymers were separated from the polymerization solution by stripping with water vapor and hot water, followed by drying in an oven at a temperature of 60 °C under reduced pressure. The molecular weight of thermoplastic elastomers collected during synthesis was measured by gel permeation chromatography (GPC); it can be found in the supplementary information (Table S1).

### 2.2. Post-Consumer Recycled Polypropylene (rPP)

Post-consumer recycled polypropylene (rPP) was provided by a Romanian collector, based on a selective waste collection system (post-user boxes manufactured by injection molding technology). It was characterized in our laboratories and had the following properties: density—1.0158 g/cm^3^, melt flow index (230 °C, 2.16 kg)—9.07 g/10 min, tensile strength of break—26.98 MPa, elongation at break—30%, notched IZOD impact strength (at 23 °C)—0.95 kJ/m^2^, VICAT softening temperature—136 °C, heat deflection temperature (HDT)—68 °C and Shore hardness—63 Sh D. It contained additives from initial processing and polyethylene (PE) coming from the collection system.

### 2.3. Glass Bubbles (GB)

Glass bubbles S22 (GB), is a powder fine dispersed, that has soda lime borosilicate glass particles with an average diameter of 16–65 μm [42], provided by 3M™ Glass Bubbles, Minneapolis, MN, USA. According to the technical sheet of the manufacturer, these glass bubbles are an alternative to conventional inorganic fillers such as silicas, calcium carbonate, talc, clay, and so forth, for many demanding applications. Also, the GB has a neutral impact on the carbon footprint of finished plastic parts.

### 2.4. Processing of Post-Consumer Recycled Polypropylene Composites

The combined effect of SBS or SIS, and GB on the mechanical and thermal properties of post-consumer recycled polypropylene was investigated by the developing of six formulations in a Brabender Plastograph at a temperature of 180 °C, mixed for 10 min with 50 rpm. The content of thermoplastic elastomers (SBS and SIS, respectively) was 10 wt% in relation with polymeric matrix, while the glass bubbles were used as 5 vol.% and 20 vol.%, respectively. The rotor speed of 50 rpm during processing of composites was selected in order to minimize exposure to high shear processes leading to breakage of glass bubbles. The compositions of post-consumer rPP/SBS/GB and rPP/SIS/GB composites are shown in Table 1.

The thermodynamic stability for all samples during melt allowing was greater than 10 min, so the formulations can be considered stable during processing. Several sheets with length of 150 mm, width of 150 mm and thickness of 4 mm and 1 mm respectively, and films of maximum 0.1 mm thickness were obtained by compression molding of melted samples using a lab hydraulic press. The pressing parameters are mentioned in Table 2.

Test specimens for the mechanical properties measurement (density, VICAT, HDT, hardness, tensile properties, Izod, DMA) of post-consumer rPP composites were obtained from pressed sheets. The post-consumer rPP was processed in the same conditions and used as reference for further characterizations.

### 2.5. Investigated Methods

#### 2.5.1. Melt Processing Characteristics

The laboratory data obtained from torque-time curves recorded on the Brabender Plastograph (Duisburg, Germany) could be used to supply the data needed for industrial extrusion processing so that the technological process would be as economical as possible. The processability behavior of rPP composites during melting in the Brabender Plastograph was evaluated by means of torque (TQ), melt viscosity (*η*) and power consumption (P) from torque-time curves according to Equations (1) and (2) [43]:

TQ = C_0_S^a^, (1)

where TQ is the torque, S is the rotor speed (rpm), C_0_ and a are constants.

(2) $$\zeta =K\dot{\gamma^{n}},$$

where *ζ* is the shear stress, $\dot{\gamma }$ is the shear rate, and K and n are the power law parameters.

Based on the Equations (1) and (2) it can be assumed that the TQ is corresponded to the *ζ*, while the S (rpm) is fitted with to the $\dot{\gamma }$. Thus, the melt viscosity (η) that is the ratio between shear stress and shear rate, here can be obtained from the ratio between torque and rotor speed Equation (3) [44]:

η = K(TQ/S),(3)

where K is a constant.

Power consumption (P) is obtained using Equation (4) [44]:

P = TQ × ω,(4)

where P is the power (W), TQ is the torque (Nm), ω is the input shaft rotation (2 πS/60).

#### 2.5.2. Optical Microscopy

Optical microscopy of the samples was evaluated using a Leica DM 2500 M microscope (Leica Camera AG, Wetzlar, Germany) equipped with a digital camera at 500× magnification.

#### 2.5.3. SEM Analysis

The morphology of the rPP and rPP composites was examined with a scanning electron microscopy (SEM) using a QUANTA 450 FEG scanning electron microscope (FEI, Eindhoven, The Netherlands), with Low vacuum Secondary Electron (LFD) Detector (500×, FEI, Eindhoven, The Netherlands).

#### 2.5.4. FTIR Study

The functional groups of rPP compounds were detected with Fourier Transform Infrared Spectroscopy in Attenuated Total Reflectance mode (ATR-FT-IR) (INTERSPEC 200-X Spectrophotometer, Interspectrum, Tartumaa, Estonia), in the spectral region of 4000–750 cm^−1^. Twenty scans were collected for each film sample at a spectral resolution of 2 cm^−1^.

#### 2.5.5. Thermal Characterization

DSC analysis was performed by placing ~8 mg from each sample in sealed alumina pans and introducing a DSC 823^e^ STARe instrument (Mettler Toledo, Greifensee, Switzerland). The test was carried out at 10 °C/min scanning speed, in a range of temperatures from ambient to 200 °C. The first heating run was made in order to remove the thermal history of the polymer. Melting enthalpy (ΔH_m_) and associated melting temperature (T_m_) were estimated from the recorded thermograms in the second heating run at 10 °C/min rate, while the crystallization peak (T_c_) was evaluated from the first cooling step at 10 °C/min rate. The degree of crystallinity of samples (**X**_c_) was calculated as the ratio between the melting enthalpy of the sample to the theoretical 100% crystallization of α-PP (209 J/g) [25].

TGA analysis of samples was performed with a TGA Q5000IR equipment (TA Instruments, New Castle, DE, USA), according to the Hi-Res MTGA method—sensitivity 1, modulate ± 3 °C for 120 s, with a speed of 10 °C/min, to obtain the maxim temperature (T_max_) and weight reduction at three heating steps, from room temperature to 310 °C, 310–560 °C, and 570–700 °C, the residue % at 700 °C, as well as the onset temperature of decomposition and mass loss, under nitrogen using a flow rate of about 50 mL/min.

VICAT softening temperature was carried out in accordance with EN ISO 306 (10 N load and the oil bath raised with 50 °C/h ± 1 °C/h (HDT-VICAT equipment, Ceast, Italy). The method consists of determining the temperature at which a penetration standard, which is subjected to a predetermined load, is 1 mm into the surface of the plastic when the temperature rises at a constant speed.

The heat deflection temperature (HDT) method consists of determining the temperature at which a predetermined arrow of 0.32 mm is obtained when the standardized test pieces were applied to a bending effort of 0.45 MPa with 120 °C/h ± 10 °C/h heating speed (EN ISO 75-1).

#### 2.5.6. Mechanical Characterization

An AS 220/X Radwag balance with density kit (Radom, Poland) was used to measure the density for all samples in ethanol by Archimedes’ principle. An average of three replicates was done for each investigated sample, at a temperature of 23 °C ± 1 °C.

Tensile properties (strain and stress) were measured using the Universal Traction Machine (Instron 8802, Norwood, MA, USA). Test specimens were used with the following dimensions—gage length 90 mm, width 6.6 mm, thickness 4 mm and a specimen pull off speed of 50 mm/min. Five samples were measured for each blend, and the average and standard deviation values were reported (ISO 527).

Hardness measurements were carried out using a Shore D durometer (ZwickRoell, Ulm, Germany), in accordance with ISO 868. Test specimens of 4 mm thickness and a loading force of 4.536 kg were used. Five test pieces were measured for each composition, and the average and standard deviation values were reported.

An Izod impact tester (Ceast, Italy), with a hammer of 2 J, was used to measure the notched Izod impact at room temperature, according to ISO 180. Five rectangular test specimens with a length of 80 mm, width of 10 mm, height of 4 mm and V-notched were used for each formulation to obtain Izod strength and their average and standard deviation were reported.

The viscoelastic properties (storage and loss modulus (*E′ and E″*) and loss factor (*tan δ*)) of post-consumer rPP samples as a function of temperature were determined using a DMA Q800 equipment (TA Instruments Inc., DE, USA) with Universal Analysis 2000 software (Version 4.5 Build 4.5.0.5, DE, USA) for the results processing. The investigated blends were examined in dual cantilever bending. The testing program of rectangular specimens (width of 10.56 mm, thickness of 3.89 mm, and length of 60 mm) was conducted from ambient temperature to 155 °C, with a constant heating scan of 3 °C/min, a frequency of 1 Hz and an amplitude of 15 μm.

The tests were investigated at 23 °C and ~50% humidity.

## 3. Results and Discussion

### 3.1. Processing Behavior of Post-Consumer rPP Blends

The melt processability of post-consumer rPP loaded with thermoplastic elastomers and glass bubbles is investigated by means of torque (TQ), melt viscosity (η) and power (P) measurements from torque-time curves—Table 3.

By adding thermoplastic elastomers to the post-consumer rPP matrix, the final torque (TQ) recorded by post-consumer rPP/SBS and rPP/SIS composites increased from 30 Nm (reached by rPP) to 36 Nm and 35 Nm, respectively. This effect can be noted due to the increased interfacial interactions ocurring at the compounding of the polypropylene matrix with elastomeric blocks [38]. It is assumed that the increase in viscosity is due to the immiscible styrene block from SIS or SBS added to the rPP matrix [38]. As the two amounts of glass bubbles were introduced in compatibilized post-consumer rPP, a general decrease of torque values is recorded. The torque decreased by 17% in the case of post-consumer rPP/SIS/GB 20 composite compared with post-consumer rPP/SIS, and 11% in the case of post-consumer/rPP/SBS/GB20 composite compared with post-consumer rPP/SBS. Accordingly, the melt viscosity (η) and power consumption (P) for the processing of rPP composites decreased compared with neat post-consumer rPP—Table 3. The recorded processing parameters highlight the effect of glass bubbles to improve the flow and melt processing of compatibilized post-consumer rPP. The similar decrease of flow resistance was reported in the case of PP/glass beads processing [2].

### 3.2. Optical Microscopy

**Figure 2:**
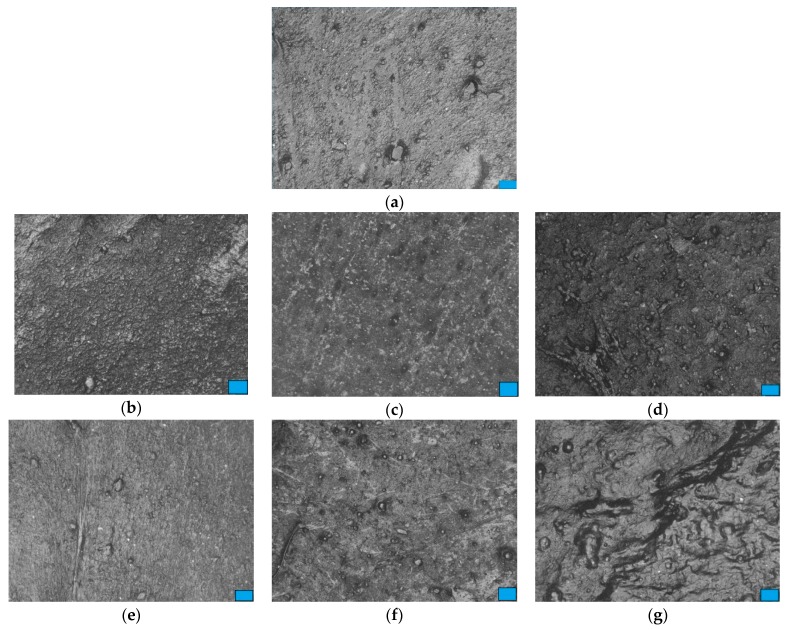
Optical microscopic images for post-consumer rPP loaded with styrene-butadiene-styrene/styrene-isoprene-styrene block-copolymers and glass bubbles: (**a**) rPP (scale bar 200 µm); (**b**) rPP/SBS (scale bar 130 µm); (**c**) RPP/SBS/GB 5 (scale bar 200 µm); (**d**) rPP/SBS/GB 20 (scale bar 100 µm); (**e**) rPP/SIS (scale bar 50 µm); (**f**) rPP/SIS/GB 5 (scale bar 100 µm); (**g**) rPP/SIS/GB 20 (scale bar 50 µm).

Figure 2 shows the optical microscopic images for post-consumer rPP composites.

Optical microscopic images for post-consumer rPP show an inhomogeneous surface (Figure 2a). A good dispersion of the continuous phase of the post-consumer rPP polymeric matrix and that of discontinuous elastomer could be observed in the rPP/SBS composite (Figure 2b). The efficiency modification by melt alloying of post-consumer rPP composites depends on the degree dispersion of styrene-diene block copolymers and glass bubbles in the polyolefin matrix, molecular weight, nature and composition of block copolymers, as well as the aspect ratio of the reinforcing agent. From Figure 2, it can be observed that the spherical glass bubbles are encapsulated into melted elastomers and rPP, leading to a high interfacial adhesion with polybutadiene and polyisoprene blocks. The density of encapsulated reinforcing agent in the post-consumer rPP compatibilized with thermoplastic elastomers increases by a loading of 20% GB in composites. It is expected that the interfacial interactions between elastomers, reinforcing agent and polymeric matrix will be beneficial for the improvement of rPP composites’ properties. The encapsulated filler was also reported in the case of ternary PP composites containing ethylene–vinyl acetate (EVA) elastomer and calcium carbonate [45]. The authors explained the high affinity for filler due to the high polarity of EVA.

### 3.3. SEM Examination

**Figure 3:**
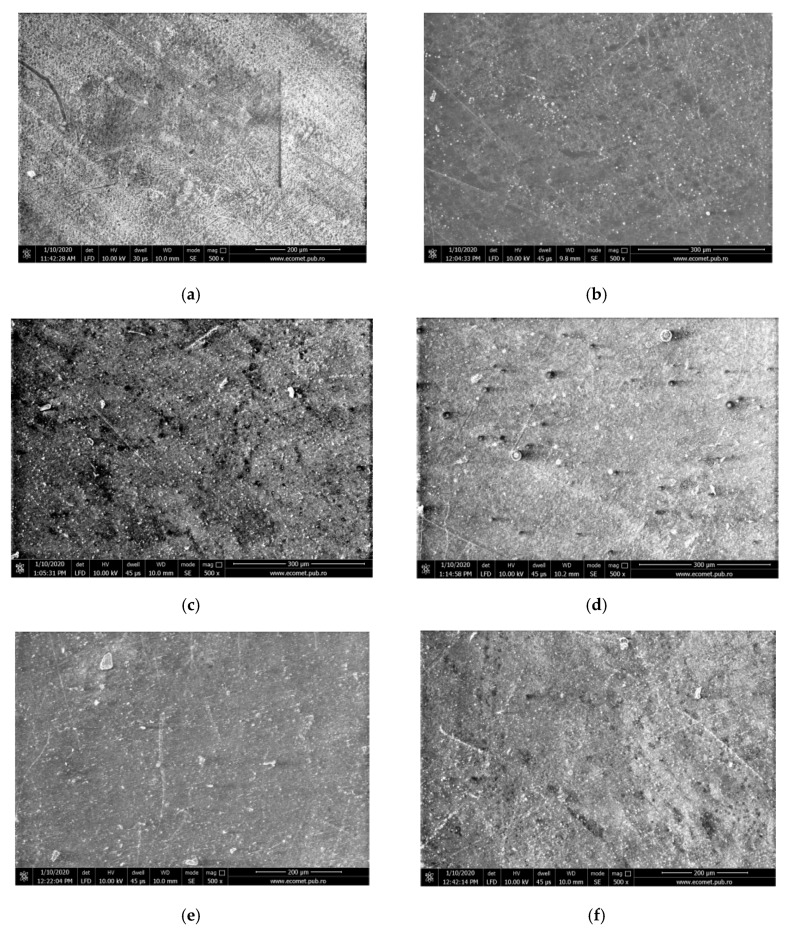

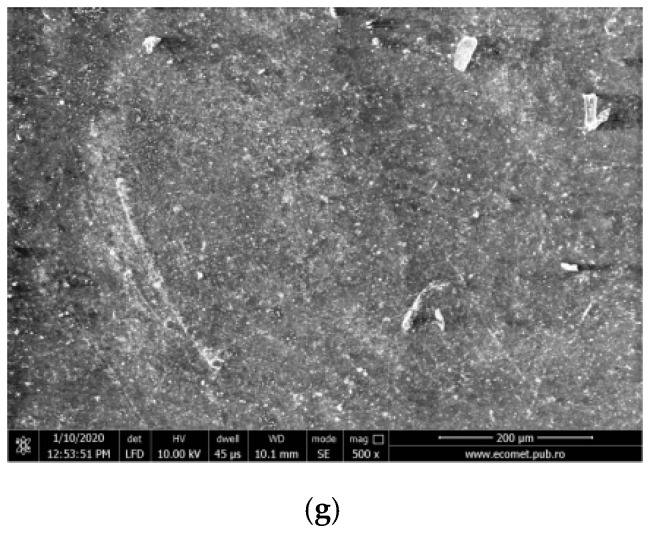
Scanning electron microscope (SEM) images for post-consumer rPP loaded with styrene-butadiene-styrene/styrene-isoprene-styrene block-copolymers and glass bubbles: (**a**) rPP (scale bar 200 µm); (**b**) rPP/SBS (scale bar 300 µm); (**c**) rPP/SBS/GB 5 (scale bar 300 µm); (**d**) rPP/SBS/GB 20 (scale bar 300 µm); (**e**) rPP/SIS (scale bar 200 µm); (**f**) rPP/SIS/GB 5 (scale bar 200 µm); (**g**) rPP/SIS/GB 20 (scale bar 200 µm).

The SEM analysis performed on the neat rPP, compatibilized rPP with SBS or SIS block copolymers and composites containing GB in 5% and 20% confirm the presence of impurities provided from the secondary recycling of PP waste and the non-homogeneous surfaces denoting the incompatibility between elastomers and the polymeric matrix (Figure 3). From Figure 3b,c, the SBS elastomer is observed as small and dispersed domains in the continuous polymeric phase. Contrary to optical images from Figure 2b, the good dispersion of elastomer can be observed in the case of SIS (Figure 3e). The GB are evidenced as encapsulated filler inside melted samples (Figure 3c,f,g) or at the surface of composite (Figure 3d).

### 3.4. FT-IR Analysis

**Figure 4:**
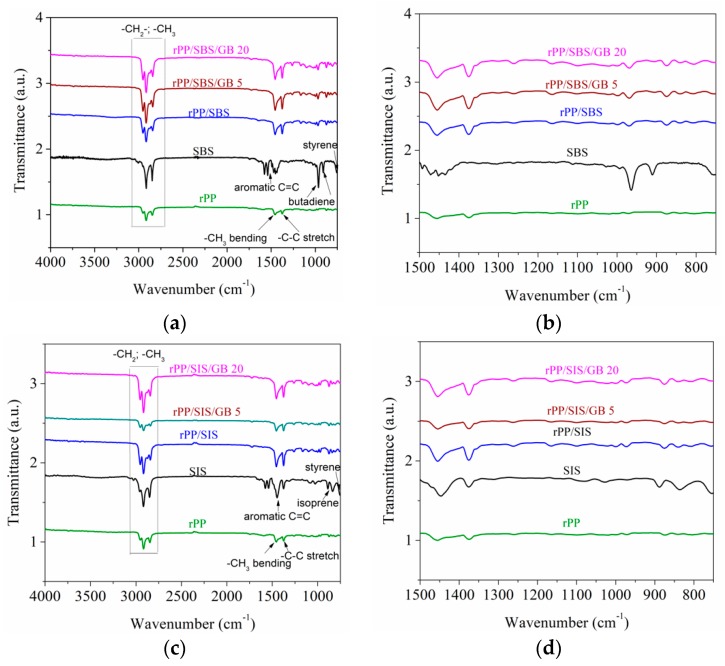
Normalized Fourier Transform Infrared Spectroscopy in Attenuated Total Reflectance mode (ATR-FT-IR) spectra for (**a**) rPP/SBS/GB composites in the spectral range 4000–750 cm^−1^, (**b**) rPP/SBS composites in the spectral range 1500–750 cm^−1^, (**c**) rPP/SIS/GB composites in the spectral range 4000–750 cm^−1^, and (**d**) rPP/SIS/GB composites in the spectral range 1500–750 cm^−1^.

The effect of elastomers and glass bubbles on the FT-IR spectra of post-consumer rPP composites is presented in Figure 4.

The spectral bands of post-consumer rPP are observed at 756 cm^−1^, 840 cm^−1^ (deformation vibrations of CH groups) [46], 874 cm^−1^ (CaCO_3_), 1020 cm^−1^ (clay), 997 cm^−1^ (-CH_2_-, -CH_3_ rocking) [46], 970 cm^−1^ (C–C stretching, CH_2_, CH_3_ rocking, amorphous) [46,47], 1455 cm^−1^ (-CH_3_ bending vibration), 1374 cm^−1^ (C-H stretching band for -CH_3_), and in the region of 2846–2952 cm^−1^ (unsaturated carbon from CH_2_ and CH_3_ groups). The absence of peak at 1730 cm^−1^ specific to C=O vibrations from saturated aldehydes indicates that the post-consumer recycled PP is not degraded.

Infrared spectra of SBS elastomer (Figure 4a) and SIS elastomer (Figure 4c) show the adsorption bands at 749–753 cm^−1^ (bending vibration of aromatic =C-H and C=C groups of polystyrene), 910 cm^−1^ (=C-H group of butadiene), 960 cm^−1^ (-CH=CH- group of butadiene), 835 cm^−1^ and 887 cm^−1^ (isoprene) [48,49], in the 1576–1375 cm^−1^ region (stretching vibration of aromatic C=C bond), 2848 cm^−1^ (-CH_2_- group), and 2915 cm^−1^ (-CH_3_ group).

Introduction of elastomers and GB into post-consumer rPP blends leads to some interactions with the rPP, facilitating interfacial adhesion increasing. Thus, small spectral changes presented in the FTIR spectrum for the post-consumer rPP/SBS/GB20 blend consist in the decrease of intensity of bands at 960 cm^−1^, and 1374 cm^−1^ compared to those of post-consumer rPP/SBS/GB5 blend. A decrease of the adsorption bands at 753 cm^−1^, 835 cm^−1^, 1375 cm^−1^, 1446 cm^−1^, 2800–3026 cm^−1^ was observed for rPP/SIS/GB 5 composite as compared with the rPP/SIS and rPP/SIS/GB 20 composites.

Based on the intensity ratio between crystalline spectral bands at A_998_ cm^−1^/A_974_ cm^−1^ [50], the degree of crystallinity of post-consumer rPP blends was estimated and is presented in the supplementary information (Table S2). The presence of the elastomer disturbs the formation of the crystalline network; consequently the degree of crystallinity decreased accordingly. Additionally, the presence of GB further reduced the crystallinity due to the dilution effect of the polymer matrix.

### 3.5. DSC Measurement

**Figure 5:**
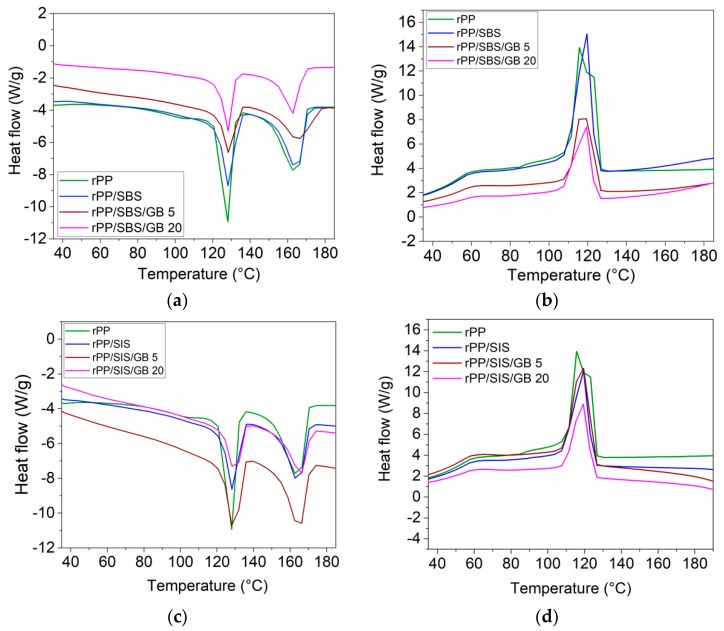
Differential scanning calorimetry (DSC) curves for rPP/SBS/GB and rPP/SIS/GB composites: (**a**,**b**) Second heating run; (**c**,**d**) Re-crystallization during cooling.

DSC curves for post-consumer rPP loaded with SIS and SBS elastomer and glass bubbles in comparison with unmodified post-consumer rPP were collected as the second heating scan (Figure 5a–d).

The thermal parameter values (melting temperature (Tm), enthalpy of melting (**∆**Hm), crystallization temperature (Tc)), as well as the degree of crystallinity (Xc) for the samples evaluated from DSC curves, are detailed in Table 4.

Two melting endothermic peaks at ~129 °C and ~164 °C, respectively, are observed during the heating process of rPP composites (Figure 5 and Table 4). The lower melting peak is attributed to the melting of the high-density polyethylene (PEHD) contamination component, whereas at higher temperature it is assigned to the melting process of the polypropylene (PP) component. Other authors also reported a melting peak around 130 °C when carrying out the thermal analysis by DSC for post-consumer recycled PP, and explained this was due to the HDPE presence in packaging films (extrusion technology) [1,34] or as bottle caps (injection-molded PE-HD) [51]. The degree of crystallinity for polyethylene (PE) was calculated based on the standard melting enthalpy corresponding to 100% crystallization of PE (290 J/g) [11]. Based on the hypothesis that the crystalline phases from PE are the same as the amorphous ones [51], 20.2% PE was found in the recycled PP.

By the addition of SIS elastomer and glass bubbles to post-consumer rPP, a slow increase in the melting temperature (T_m_) of blends is observed, due to the reinforcing effect of elastomer and GB. The melting temperature (T_m_) for rPP/SBS/GB 20 composite evidenced a decrease with ~2 °C less than that of the compatibilized sample, due to the good dispersion of butadiene blocks in the amorphous phase of rPP as well as to the encapsulated GB inducing a lower melting temperature. The same decrease was observed at DSC analysis of random PP modified with commercial SBS copolymer [14]. The post-consumer rPP/SIS blend shows a degree of crystallinity of 17.0%, very close to that of post-consumer rPP (17.7%), while post-consumer rPP/SBS blend shows a degree of crystallinity of 16.0%. It is assumed that the presence of isoprene blocks in the SIS structure created a high interfacial compatibility with rPP polymeric matrix without disturbing the arrangement of the crystal lattice. In the case of SBS, the disruption of the lattice structure of rPP taking place by reduction of the active centers of crystallization, with the decrease in crystallinity. The SIS compatibilizer is more entangled than SBS and the crystallization rate is slower. The introduction of glass bubbles into compatibilized post-consumer rPP, due to the high aspect ratio and flow in melt processing (Table 3), reduced the growth rate of spherulites. As a consequence, the crystallinity of rPP composites decreased. The maximum lowering effect of the degree of crystallinity is generated by rPP composite with SBS and 20% GB (X_c_ 15.6%), which shows the most appropriate melt viscosity with the recycled polypropylene (Table 3). Other authors showed that the degree of crystallinity of polyolefin samples raised by the addition of elastomer (SEBS) [52], glass fibers [53] and talc [21], while the introduction of montmorillonite clay created a decrease in crystallinity [54]. It is possible that the agglomeration of filler in melted samples to contribute to the crystallinity decreasing. Regarding the crystallization temperature (T_c_) of composites, those containing 5% GB exhibit higher T_c_ values (121.07 °C) compared with that for rPP loaded with elastomer (120.6 °C), in good agreement with the stiffness increasing (Figure S1). A lower T_c_ value for the post-consumer blends containing 20% GB than those of neat rPP and compatibilized blends is recorded, potentially due to a plasticizing effect of SBS and SIS elastomers. However, the most changes in thermal properties are provided by rPP/SBS/GB 20 composite.

### 3.6. TGA

**Figure 6:**
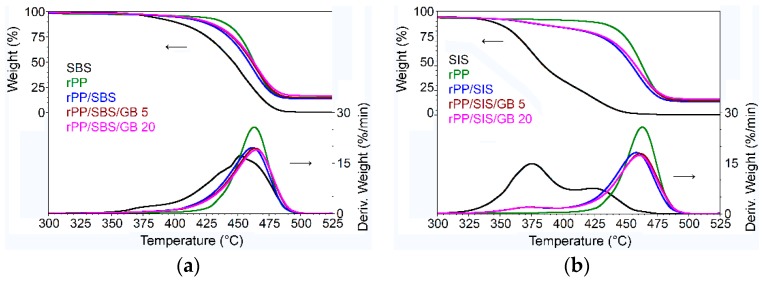
Thermogravimetric analysis (TGA) curves for: (**a**) post-consumer rPP/SBS and post-consumer rPP/SBS/GB composites compared with post-consumer rPP and SBS; (**b**) post-consumer rPP/SIS and post-consumer rPP/SIS/GB composites compared with post-consumer rPP and SIS.

The thermal stability of rPP/SBS/GB, rPP/SIS/GB composites, post-consumer neat rPP, SIS, and SBS was determined by TGA—Figure 6a,b.

Table 5 illustrates the degradation temperatures and weight loss for obtained composites, SIS and SBS elastomers.

The post-consumer rPP showed an onset at degradation temperature (T_i_) of 445.3 °C, a weight loss of 96.01% and a residue in a nitrogen atmosphere at 700 °C of 9.68%, which means the presence of specific processing additives such as talc, clay, calcium carbonate, and so forth. The presence of elastomer (SBS or SIS) and glass bubbles in the post-consumer rPP composites has no significant marks on their thermal stability until 310 °C. The weight loss of 1.26%–1.62% recorded up to 310 °C is proof that the developed composites are not degraded during melt processing (Table 5), so their thermal stability is very good.

TG analysis in the range of 310–560 °C evidenced the oxidation of carbon [15], as the main decomposition step, where all composites lost more than 82% of the mass. DTG curves for elastomers (Figure 6) showed two prominent peaks around 375.3 °C and 423.6 °C for SIS indicating a weight loss of 98.63% and one small peak at 452.3 °C assigned to SBS corresponding to a weight loss of 99.26%. The incorporation of SBS into post-consumer rPP matrix led to a decrease in its thermal stability with 2 °C. Contrary to our study, by introduction of poly (ethylene-co-methacrylic acid) copolymer compatibilizer into polyolefin waste, the increased thermal stability of blend was reported [55]. From Table 5 it could be observed that, at any step of the investigated temperatures, the presence of glass bubbles increased the thermal stability of post-consumer rPP composites with ~3 °C, while the mass loss decreased. The glass bubbles can act by blocking the moving of thermal decomposition products from rPP composites, as well as by their absorption effect [23]. It demonstrated that the thermal insulation effect of glass bubbles. An increase in the maximum temperature at which the degradation occurred (T_max_) by 5.36 °C is reported for PP composites with 1.5 wt% TiO_2_ nanoparticles [56], as in the case of glass fibers’ incorporation in the polyethylene matrix [53]. The weight losses as residue in a nitrogen atmosphere at 700 °C were ~9% for compatibilized post-consumer rPP, ~9.5% and ~11.7% for rPP composites containing 5% and 20%, respectively, proportional to the amount of elastomer and filler from the rPP composite production.

### 3.7. Mechanical Properties

The influence of elastomer type and the content of glass bubbles on the mechanical properties of post-consumer rPP composites, such as density, tensile strength, elongation at break, hardness, VICAT temperature point, HDT and IZOD impact, were studied (Table 6).

**Figure 7:**
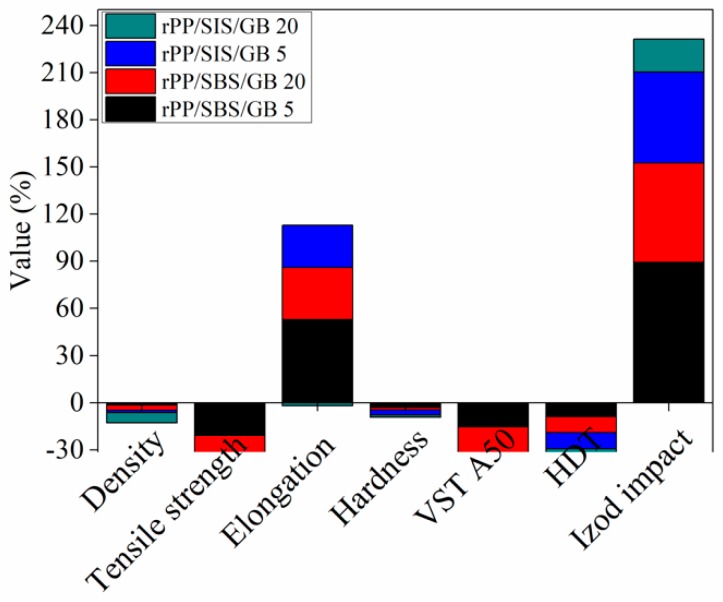
Variation in the mechanical properties of rPP composites containing SIS, SBS, and GB versus rPP.

The influence of the elastomer nature and the amount of glass bubbles required to modify the mechanical properties of rPP are plotted in Figure 7.

Depending on the nature and elastomer and loading of glass bubbles into rPP, different materials with specific mechanical properties were obtained. As observed from Figure 7, the concomitant improvement on density, flexibility and IZOD impact, without declining more properties, is obtained. These effects are assigned to the GB, which amplifies the elastomer role in composites. The glass bubble from composites has an effect of decreasing the density of post-consumer rPP composites. Thus, the density of post-consumer rPP is diminished from 1.0158 g/cm^3^ to 1.0007 g/cm^3^ and 0.9826 g/cm^3^ in the case of using 10% SBS, 5% and 20% glass bubbles, respectively. Compared with rPP, at 20%, glass bubbles loading, a weight reduction with 3.2% in the case of rPP/SBS composite and ~7% in the case of rPP/SIS composite, respectively, is obtained. This behavior can be explained by the high surface area to volume ratio of GB, allowing the use of low quantities of raw materials’ demand in a variety of applications where the light items are desired. The development of light plastic components is of real interest to auto components [57], aerospace, mobile devices, sport applications. Also, by using low quantities of reinforcing agent, low petrochemical resources are consumed, resulting in environmental sustainability by offering lower material costs and reduced energy requirements. It is expected that, without elastomer, the GB produces only an increase in the density and melt viscosity of rPP composites, while the elongation and tensile strength decreased significantly.

The appreciable improvement in the performance properties of rPP composites is observed at the impact strength values. Compared with the notched Izod impact of neat rPP, the rPP/SBS/GB composites show that the impact strength increased by 89% and 63%, while the rPP/SIS/GB composites contribute to a 57% and 21% increase, according to the content of glass fibers—Figure 7. This increase is the consequence of interfacial adhesion between the components of post-consumer rPP blends. Similarly, other authors reported high elasticity and notched impact IZOD of post-consumer rPP compounded with elastomers, compared with unmodified PP wastes [37].

Thus, at 10% content of elastomer (SIS or SBS) the tensile strength of rPP/SBS composite decreased with 18.5%, and with 17% in the case of rPP/SIS composite compared with neat rPP—Table 6. The reduction in tensile strength property was also reported for virgin PP/elastomer blends and PP waste/elastomer blends [11,58,59]. The authors explained this behavior was due to the incapability of elastomer and polypropylene to form a homogenous phase, and the low strength amorphous rubbery matrix. With the introduction of glass bubbles into the composites, a small increase in the tensile property of rPP/SIS and rPP/SBS composites is observed, in good correlation with the reduction in crystallinity (Table 4) and the stiffness (Figure S1). On the contrary, elongation at break considerably increased in comparison with unmodified post-consumer rPP, due to the elastomer effect that acted as a plasticizer allowing better material stretching [39]. The post-consumer rPP/SBS blend exhibited a higher elongation at break (72.22% ± 9.46%) than that of post-consumer rPP/SIS blend (55.20% ± 13.5%) (Table 6). This is due to the low viscosity of SIS, which showed an increased crystallinity than SBS, leading to the reduction in elongation, while the tensile strength is increased. The introduction of elastomer in the post-consumer rPP matrix offers the possibility to diminish the Shore hardness. The obtained rPP composites showed VICAT softening temperature (VST) values lower (110–115 °C) than those of neat rPP (136 °C) and rPP loaded with elastomer (~130 °C), due to both the presence of HDPE in neat rPP (as confirmed by DSC analysis) and glass bubbles that show a limited adherence to the polyolefin matrix. The behavior at heat deflection temperature (HDT) follows the same rule as the VICAT test. No significant differences regarding the VST and HDT measurements of rPP composites were obtained.

**Figure 8:**
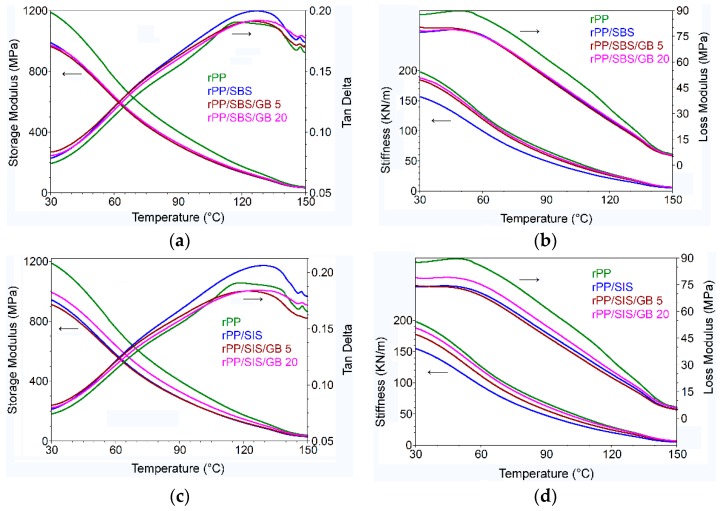
Dynamic mechanical analysis (DMA) analysis. Storage modulus and tan δ for: (**a**) post-consumer rPP/SBS/GB and (**c**) post-consumer rPP/SIS/GB; Loss Modulus and stiffness for: (**b**) post-consumer rPP/SBS/GB and (**d**) post-consumer rPP/SIS/GB.

The effect of glass bubbles and thermoplastic elastomers on the visco-elastic behavior of post-consumer recycled PP was investigated by DMA, in the region of 30–150 °C. Figure 8a–d shows the storage modulus (*E′*) and *tan δ* (loss factor) and loss modulus and stiffness versus temperature for composites prepared with SBS and SIS and reinforcing agent, respectively.

The incorporation of the synthesized elastomers in the rPP phase led to the decrease of storage modulus (*E′*) and stiffness. From storage modulus-temperature curve (Figure 8a,c) it can be observed that at 90 °C (rubbery region), the post-consumer rPP/SIS shows the lower storage modulus (289.3 MPa) in comparison with the post-consumer rPP/SBS blend (313.4 MPa). This is supported by the entanglements of polybutadiene from SBS that are higher than those of polyisoprene from SIS. This trend is observed at each temperature test—supplementary material—Table S3. The storage modulus value in the rubbery region denotes the ability of macromolecules to resist the intermolecular slippage. The decrease of storage modulus with the introduction of block copolymer are in line with previous papers [15,52]. With the incorporation of GB 5% (vol.) no modification in the storage modulus of the post-consumer rPP composites is observed, while in the case of 20% GB an increase of ~14% and ~3% for post-consumer rPP/SIS and rPP/SBS blends, respectively, was observed. GB has a higher reinforcing effect on polybutadiene, leading to an increase of the deformation effect manifested by the increased storage modulus.

Figure 8a,c displays the dependence loss factor (*tan δ*)—temperature of samples. *Tan δ* represents the loss modulus divided by the storage modulus. Usually, the high *tan δ* results are in agreement with viscous behavior, whereas low *tan δ* results are correlated with elastic behavior [26]. *Tan δ* values increased with the introduction of compatibilizers into the post-consumer rPP matrix. It reached its maximum at 127.31 °C (0.1997 MPa) and 128.5 °C (0.2064 MPa) for post-consumer rPP/SBS and rPP/SIS blends, respectively. This reflects the viscous nature of post-consumer rPP/elastomers, in good agreement with the melt viscosity reported in Table 3. With the incorporation of glass bubbles, a decrease in the *tan δ* values is observed for both compatibilizers used in this study. The post-consumer rPP/SIS/GB composites show that the *tan δ* values were lower than those of post-consumer rPP/SIS/GB composites. A new notable loss peak at ~50 °C was recorded by the rPP/elastomer/GB composites. It is linked with the chain mobility of rPP immobilized on the GB surface. With the incorporation of glass bubbles into post-consumer rPP compounds a significant increase of stiffness is observed at tested temperatures (30 °C, 90 °C, and 120 °C) (Figure S1). This behavior favors the dimensional stability of plastic items. The performance properties of rPP modified with thermoplastic elastomers and glass bubbles such as reducing the dimensions, improving the tensile, elongation, impact strength, thermal stability, as well as melt processing, sustain the use of these formulations for the engineering applications.

## 4. Conclusions

These results may point out that the properties of recycled PP composites modified with two thermoplastic elastomers with certain molecular mass can be enhanced significantly through the addition of glass bubbles with the aim of use in engineering applications.

The reinforcing mechanism of post-consumer rPP is related to the morphology of block copolymers and the content of glass bubbles used. The introduction of thermoplastic elastomers in rPP blends led to a significant increase in flexibility and Izod impact, while by adding GB, the processability, thermal stability, weight reduction, storage modulus, loss modulus, and stiffness of blends increased.

## Figures and Tables

**Table 1:** Compositions for post-consumer recycled polypropylene (rPP) compounds.

Sample	rPP [wt%]	Thermoplastic Elastomer [wt%]	GB [vol.%]
rPP	100	-	-
rPP/SBS	90	10	-
rPP/SBS/GB 5	89.1	9.9	5
rPP/SBS/GB 20	86.7	9.6	20
rPP/SIS	90	10	-
rPP/SIS/GB 5	89.1	9.9	5
rPP/SIS/GB 20	86.7	9.6	20

**Table 2:** Processing parameters for obtaining of post-consumer rPP/SBS, rPP/SIS, rPP/SBS/GB, and rPP/SIS/GB sheets and films.

Parameter	U.M.	Value	
		Film	Sheet
Preheating time	min	2	4
Pressing time	min	2	2
Pressing temperature	°C	185	185
Pressure	atm	150	125
Cooling time	min	45	45

**Table 3:** Final torque (TQ), melt viscosity (η), and power consumption (P) registered at processing of post-consumer rPP and its composites at 180 °C and 50 rpm.

Sample	Torque, TQ (Nm)	Melt Viscosity, η (Nm/rpm)	Power, P (kW)
rPP	30	0.6	0.157
rPP/SBS	36	0.72	0.188
rPP/SBS/GB 5	35	0.7	0.183
rPP/SBS/GB 20	32	0.64	0.167
rPP/SIS	35	0.7	0.183
rPP/SIS/GB 5	34	0.68	0.177
rPP/SIS/GB 20	29	0.58	0.151

**Table 4:** DSC parameters for post-consumer rPP/TPE/GB composites.

Sample	∆H_m,PE_ (J/g)	T_m,PE_ (°C)	∆H_m,PP_ (J/g)	T_m,PP_ (°C)	T_c,PP_ (°C)	T_c,PE_ (°C)	X_c,PE_ (%)	X_c,PP_ (%)
rPP	29.5	127.9	33.8	164.6	121.70	115.69	10.1	17.7
rPP/SBS	22.0	129.3	27.5	164.1	120.62	114.81	8.4	16.0
rPP/SBS/GB 5	20.2	129.5	26.9	165.4	121.07	115.13	7.7	15.8
rPP/SBS/GB 20	22.6	128.6	25.7	162.8	120.13	114.07	8.9	15.6
rPP/SIS	23.2	129.6	29.1	164.4	120.68	114.37	8.9	17.0
rPP/SIS/GB 5	23.2	129.8	28.7	165.0	121.07	114.92	8.9	16.9
rPP/SIS/GB 20	23.7	130.3	27.9	166.3	120.55	114.62	9.4	16.8

**Table 5:** Degradation temperature, weight loss, and residue for different post-consumer rPP composites.

Sample	23–310 °C	310–560 °C			560–700 °C		700 °C	Onset Degradation	
	Wt. Loss (%)	Wt. Loss (%)	T_max_._1_ (°C)	T_max.2_ (°C)	Wt. Loss (%)	T_max_._3_ (°C)	Wt. Loss (%)	T_i_ (°C)	Wt. Loss (%)
rPP	1.94	83.35	463.4	-	5.04	633.4	9.68	445.3	96.01
SBS	0.29	99.26	452.3	-	0.23	-	0.22	415.8	99.69
rPP/SBS	1.62	85.14	461.7	-	4.51	641.8	8.72	434.9	96.72
rPP/SBS/GB 5	1.28	84.46	463.7	-	4.46	645.1	9.80	437.0	97.11
rPP/SBS/GB 20	1.16	82.52	464.9	-	4.35	653.1	11.98	437.6	97.31
SIS	0.90	98.63	375.3	423.6	0.03	-	0.44	353.0	96.86
rPP/SIS	1.46	85.40	370.9	458.4	4.47	641.3	8.6	429.5	95.97
rPP/SIS/GB 5	1.35	84.67	374.7	461.5	4.51	649.9	9.47	431.8	96.17
rPP/SIS/GB 20	1.26	82.76	373.3	460.8	4.31	648.2	11.67	431.0	96.30

**Table 6:** Mechanical tests for post-consumer rPP/SBS, rPP/SIS, rPP/SBS/GB, rPP/SIS/GB composites compared with those for post-consumer rPP.

Sample	Density (at 23 °C) (g/cm^3^)	Tensile Strength at Break (MPa)	Elongation at Break (%)	Hardness (Sh D)	VST A50 (°C)	HDT (°C)	Izod Impact (kJ/m^2^)
rPP	1.0158 ± 0.0066	26.98 ± 1.50	30.30 ± 6.12	63 ± 2	136 ± 2	68 ± 1	0.95 ± 0.15
rPP/SBS	1.0180 ± 0.0021	21.99 ± 1.75	72.22 ± 9.46	60 ± 1	130 ± 2	63 ± 1.5	2.35 ± 0.34
rPP/SBS/GB 5	1.0007 ± 0.0013	21.36 ± 1.24	46.36 ± 8.40	61 ± 2	115 ± 2	62 ± 1	1.80 ± 0.21
rPP/SBS/GB 20	0.9826 ± 0.0005	20.45 ± 1.23	40.30 ± 5.80	62 ± 2	112 ± 2	61 ± 2	1.55 ± 0.17
rPP/SIS	1.0173 ± 0.0006	22.39 ± 1.45	55.20 ± 13.50	59 ± 1	128 ± 2	62 ± 1.5	1.75 ± 0.21
rPP/SIS/GB 5	1.0001 ± 0.0049	23.51 ± 1.34	38.78 ± 6.52	61 ± 2	110 ± 2	61 ± 1.5	1.50 ± 0.34
rPP/SIS/GB 20	0.9494 ± 0.0052	24.22 ± 1.63	29.69 ± 4.34	62 ± 1	115 ± 2	59 ± 1	1.15 ± 0.14

## Supplementary Materials

The following are available online at https://www.mdpi.com/1996-1944/13/3/543/s1, Figure S1. Stiffness for post-consumer rPP loaded with styrene-butadiene-styrene/styrene-isoprene-styrene block-copolymers at 30 °C, 90 °C, and 130 °C. Table S1. Molecular weight of SBS and SIS synthesized by anionic polymerization. Table S2. A_998_ cm^−1^/A_974_ cm^−1^ estimated from FTIR spectra. Table S3. Some data for loss modulus, *tan δ* and storage modulus for post-consumer rPP/SBS, rPP/SIS, rPP/SBS/GB, rPP/SIS/GB composites compared with that of post-consumer rPP.
