# Supplementary material for: Effect of Styrene-Diene Block Copolymers and Glass Bubbles on the Post-Consumer Recycled Polypropylene Properties
Source: Materials (Basel). 2020 Jan 23;13(3):543. doi: 10.3390/ma13030543 (PMC7040787; doi:10.3390/ma13030543)
Supplement: Supplementary file 1 [file materials-13-00543-s001.pdf]

materials

Supplementary Materials: Effect of Styrene-Diene Block Copolymers and Glass Bubbles on the Post-Consumer Recycled Polypropylene Properties

Maria Rapa, Bogdan Norocel Spurcaciu *, George Coman, Cristian Andi Nicolae, Augusta Raluca Gabor, Paul Niculae Ghioca, Andrei Constantin Berbecaru, Ecaterina Matei * and Cristian Predescu

Table S1. Molecular weight of SBS and SIS synthesized by anionic polymerization.

Property	U.M.	SBS	SIS
Polystyrene content	%	31.8	30.4
Molecular weight	g/mol	190,000	123,500
Polystyrene block molecular weight	g/mol	15,650	18,800
Polybutadiene block molecular weight	g/mol	128,200	-
Polyisoprene block molecular weight	g/mol	-	85,900

Table S2. A_{998} cm^{-1}/A_{974} cm^{-1} estimated from FTIR spectra.

Sample	A_{998} cm^{-1}/A_{974} cm^{-1}
rPP	0.64
rPP/SBS	0.36
rPP/SBS/GB 5	0.28
rPP/SBS/GB 20	0.18
rPP/SIS	0.30
rPP/SIS/GB 5	0.22
rPP/SIS/GB 20	0.16

Table S3. data for loss modulus, *tan δ* and storage modulus for post-consumer rPP/SBS, rPP/SIS, rPP/SBS/GB, rPP/SIS/GB composites compared with that of post-consumer rPP.

Sample	Loss Modulus (*E*"-Peak)		Tan Delta (Tan δ (max) Peak)		Storage Modulus (MPa)		
	Temperature	Height	Temperature	Height	Temperature		
	°C	MPa	°C	MPa	30 °C	90 °C	130 °C
rPP	48.85	89.87	118.55	0.1906	1187	402.9	120.9
rPP/SBS	55.93	77.28	127.31	0.1997	987.9	313.4	93.48
rPP/SBS/GB 5	51.78	78.59	124.08	0.1911	961.5	313.4	95
rPP/SBS/GB 20	54.37	77.29	127.87	0.1918	971.7	326.2	101.1
rPP/SIS	53.84	72.91	128.5	0.2064	942.3	289.3	85.18
rPP/SIS/GB 5	51.61	72.47	122.18	0.1833	911.4	287.4	88.06
rPP/SIS/GB 20	44.19	79.06	126.49	0.184	992.4	330.8	103.2

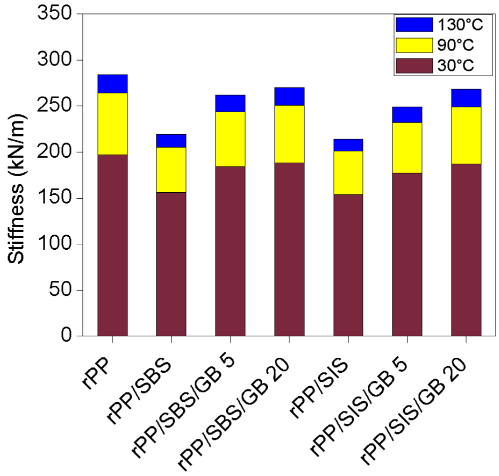

Figure S1. Stiffness for post-consumer rPP loaded with styrene-butadiene-styrene/styrene-isoprene-styrene block-copolymers at 30 °C, 90 °C, and 130 °C.

© 2020 by the authors. Licensee MDPI, Basel, Switzerland. This article is an open access article distributed under the terms and conditions of the Creative Commons Attribution (CC BY) license (http://creativecommons.org/licenses/by/4.0/).
